# Enhancing Dental Education: Impact of Remote Teaching on Dental Students’ Academic Performance in Orthodontics—A Pilot Study

**DOI:** 10.1177/23821205241293488

**Published:** 2024-11-03

**Authors:** Heidi Arponen, David P. Rice, Emma Juuri

**Affiliations:** 1 Department of Oral and Maxillofacial Diseases, 3835 University of Helsinki, Helsinki, Finland; 2 Department of Oral and Maxillofacial Diseases, Helsinki University Hospital Head and Neck Center, Helsinki, Finland; 3 Cleft Palate and Craniofacial Center, Department of Plastic Surgery, University of Helsinki and Helsinki University Hospital, Helsinki, Finland

**Keywords:** dental education, orthodontics, distance learning

## Abstract

**OBJECTIVE:**

Remote teaching strategies have been widely adapted in recent years but their impact on dental students' learning outcomes is less well understood. The aim of this mixed-method pilot study was to examine the impact of remote teaching on undergraduate dental student's learning outcome as assessed by examination performance and student feedback in an orthodontics course.

**METHODS:**

Out of the lectures (19 in total), 10 were delivered remotely (live online lecture or video recording) and 9 as traditional classroom teaching in 2023. The course examination, completed by 47 of the 48 course participants, consisted of 38 single best answer multiple choice questions with 2 questions dedicated to each lecture topic.

**RESULTS:**

The average lecture attendance was 83% of the students enrolled in the course. Statistical analysis revealed no significant association between examination scores and the mode of teaching or the mandatory nature of the lecture (*r_s_* = −0.022, *p* = .897 and *r_s_* = −0.048, *p* = .773, respectively). However, individual students’ examination scores correlated positively with the frequency of lecture attendance (*r_s_* = 0.416, *p* = .004). Students’ preferences were in favor of blended learning approach, although notable individual differences in opinions were observed in the feedback received.

**CONCLUSION:**

The findings of this study support the hypothesis that remote teaching generates an equally good learning outcome in orthodontics as classroom lectures, as reflected by examination performance.

## Introduction

The global COVID-19 pandemic rapidly changed the landscape of higher education.

In recent years higher education providers have increasingly shifted from traditional classroom teaching toward online and/or blended teaching approach. Blended learning as defined by Dziuban et al,^1^ is an instructional method that includes the efficiency and socialization opportunities of the traditional face-to-face classroom with the digitally enhanced learning possibilities of the online mode of delivery. At present, universities have adopted varied teaching strategies, with some providing solely face-to-face teaching, while others providing simultaneous remote teaching in dental education. Online education affects both students and teachers.^2^ While it presents an opportunity for digital innovations and enhanced education, it may also predispose individuals into additional stress potentially resulting in suboptimal academic performance.^3–5^ Online education can potentially promote effective learning and teaching, or present as a constraint for collaborative learning as defined by Yip and Barnes.^6^ Incorporating online education is especially challenging in the field of dentistry where theoretical education is complemented by social learning and intensive clinical practice.^7^ A previous investigation revealed that the examination results of undergraduate dental students were influenced by the remote teaching during the COVID-19 pandemic,^8^ and that this alteration is likely multicausal.

Dental students experience considerable levels of stress during their training stemming from academic workload, examinations, clinical care, faculty-related factors, and personal factors.^9,10^ Perceived stress, in turn, could impact the academic performance of individual students, though its effects seem modest when considered at a group level.^10^ Much of the existing literature concerning the effects of remote teaching on academic performance has been conducted during the pandemic, potentially influencing students’ stress level.^3,11,12^ Nevertheless, a previously published review concluded that universities providing dental education should re-evaluate their policies and curricula, and permanently incorporate appropriate methods of online learning into their teaching curriculum.^4^ In accordance with this recommendation, studies among Asian dental students and British postgraduate orthodontic students reported a favorable opinion towards blended learning.^13,14^ A permanent shift in the mode of education delivery would constitute a significant change in the curriculum, warranting an investigation into its effects on students’ learning. A successful fully online orthodontic preclinical curriculum has been presented by Liu et al.^15^ However, to the best of our knowledge, data on the effects of blended teaching approach on undergraduate dental students’ academic performance in orthodontic training are lacking. The aim of this study is to assess whether a mixed method teaching of didactic orthodontic classes enhances the student's learning and satisfaction. We hypothesize that online-delivered lectures generate learning outcomes, as assessed by examination results, that are equally good as those from classroom lectures.

## Materials and methods

The research ethics committee of the Faculty of Medicine at the University of Helsinki had approved the study (8/2023). The required sample size was estimated based on previous recommendations of 10 to 40 participants for pilot studies.^16^

### Study population

The study group comprised 48 third-year undergraduate dental students who participated in the orthodontics course at the University of Helsinki. Inclusion criterion was course participation. One student was excluded from the study due to discontinuation of the course participation. Out of the included study subjects 9 were male and 38 females.

Participation in the study was voluntary, and written information was provided at the course's onset. None of the 48 students participating in the course objected to the use of their course records and course feedback in the study. During the current study no pandemic-related or other restrictions were issued nationally or locally.

### Study design and data collection

The reporting of this study conforms to the SQUIRE-EDU publication guidelines^17^ (Supplemental file).

This pilot study examined an orthodontic course conducted during the third study year within a 4-month period spanning from August to December 2023. The course constitutes majority of the orthodontic teaching provided in the predoctoral dental education at University of Helsinki. The main topics of the course are craniofacial growth, craniofacial anomalies, etiology and classification of malocclusions, cephalometrics, cellular basis of tooth movement, managing developing dentition, fixed and removable orthodontic appliances, missing and impacted teeth in orthodontics, orthodontic first aid, and screening of malocclusions in public healthcare. The course is mandatory for all undergraduate students and includes theoretical lecture-based teaching and simulation laboratory teaching (all face-to-face sessions) facilitated by 6 experienced university lecturers. Of the 19 lectures included in the course, 9 (47%) require compulsory attendance and attendance is monitored at all teaching sessions. Before the onset of the course, every other lecture was allocated by the authors HA and EJ in chronological order into 1 of 2 groups; those delivered as classroom teaching or remote teaching on Zoom platform. An exception of the allocation pattern was made on 2 days, where several lectures were scheduled. The lecture delivery mode was organized as either remote or in-person for the whole day, to make studying more convenient. Sample allocation was balanced thereafter so that eventually 10 out of the 19 lectures were delivered remotely as a live online lecture or as a video recording of the lecture. Four of these remote lectures were mandatory. The lecture structure consisted of context and learning objectives, content, and closure. Duration of the lectures varied between 45 and 90 min. The video recorded lecture duration was 20 min. In the orthodontics course studied, the handout material distributed to the students following the lecture comprised the main points of the lecture, but additional material and images were available during each lecture. If a student missed a mandatory lecture, they would need to hand in a written assignment on the subject to verify reaching of the learning objectives of the class.

In addition to the course/module under current investigation, undergraduate teaching in orthodontics is supplemented with clinical hands-on education with patients, and further lectures, tutorials and simulation laboratory sessions. A summative final exam in orthodontics is held during the last year of dental studies.

The course examination consisted of 38 single best answer (SBA) multiple choice questions, that were consistently constructed by the authors HA and EJ in accordance with the principles outlined in The National Board of Medical Examiners^®^ item-writing manual and following the recommendations by Walsh et al.^18,19^ The examination was unvalidated for this pilot study. Each lecture was represented by 2 questions, which were aligned with the course's learning objectives. The students’ academic performance was assessed as examination result outcome.

Students’ opinions on and preferences regarding different teaching methods for enhancing learning were collected at the end of the course through an unvalidated anonymous online questionnaire. The questionnaire included 2 multichoice and one open-ended question (Table 1), designed to elicit students’ general views about remote learning. Responding to the questionnaire was optional following the University of Helsinki policy on course feedback.

**Table 1.** Opinion on and preference of different teaching methods in enhancing learning of 31 dental students.

QUESTION	CODE	THEME	NUMBER OF RESPONDENTS	SAMPLE QUOTE EXAMPLES
My learning is best supported by	Classroom teaching		2/4 (6%)	
	Remote teaching		0 (0%)	
	A combination of classroom and remote teaching		2/4 (6%)	
My academic workload is reduced by	Classroom teaching		5/31 (16%)	
	Remote teaching		8/31 (26%)	
	A combination of classroom and remote teaching		11/31 (35%)	
	I don't know		7/31 (23%)	
Other remarks	Unstructured open question	Remote teaching	3/11 (27%)	*Being able to stay home and study remotely was good*
		General positive aspects	8/11 (72%)	*Great learning atmosphere!*
		General negative aspects	5/11 (45%)	*Lots of new things to learn which made the course confusing* *I would have wished for more teaching on different orthodontic appliances*

### Statistical analysis

Statistical analyses were performed using SPSS software 23.0 (IBM Corp., NY, USA). While continuous variables (attendant numbers, examination scores) were presented as median and percentages, categorical variables were described in percentages (teaching mode, lecture attendance requirement). The Shapiro-Wilk normality test was used to determine the normal distribution of the variables. The Mann-Whitney U test and Spearman's rank correlation were conducted to evaluate difference in the examination question scores between the different teaching delivery modes and lecture attendance requirement. The association between examination scores and lecture attendance of individual students was examined with Spearman's rank correlation as a complete case analysis. A value of *p* < .05 was considered statistically significant. Observations with missing data were excluded.

## Results

**Figure 1.**
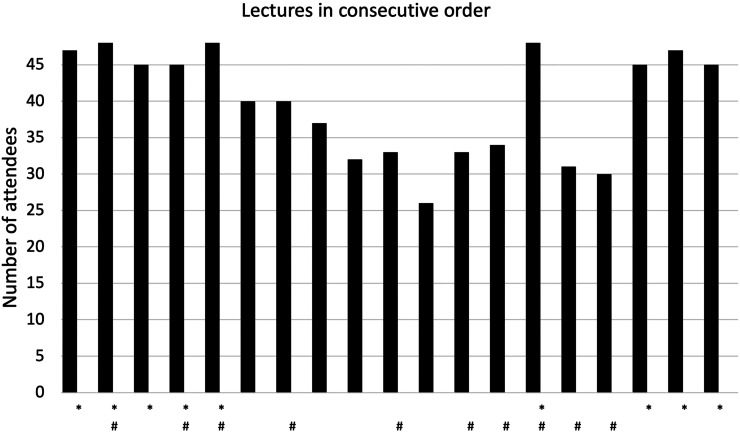
Lecture attendance for the 48 students. *Compulsory lecture. #Remote lecture.

On average, 40 out of 48 students (83%) participated in the lectures, with attendance range from 26 to 48 students (Figure 1). The average lecture attendance was 96% for the compulsory lectures and 71% for the voluntary ones. The number of students attending lectures positively correlated with the lecture attendance requirement (*r_s_* = 0.489, *p* = .033). Throughout the course, an average of 39 students participated in the classroom lectures and 38 in the remote lectures. The names of the students were missing for one nonmandatory lecture. However, the number of attendees was recorded for all the lectures. And 75% of male students and 71% of female students attended the nonmandatory remote lectures, while the nonmandatory in-person lectures were attended by 67% of male and 65% of female students. The difference between the genders in attendance was statistically insignificant.

A total of 47 students (98%) participated in the supervised digital on-site course examination. The examination utilized the Safe Examination Browser program which prohibited the use of online browsing. The examination had a maximum completion time of 3h and a one-attempt limit. One student was enrolled for but not present in the examination. The average duration of completing the examination was 41 min (range 17 to 75 min). The examination awarded one point for each correct answer in the multiple-choice questions. The average score in the examination was 33 out of 38 (range 21 to 36).

**Figure 2.**
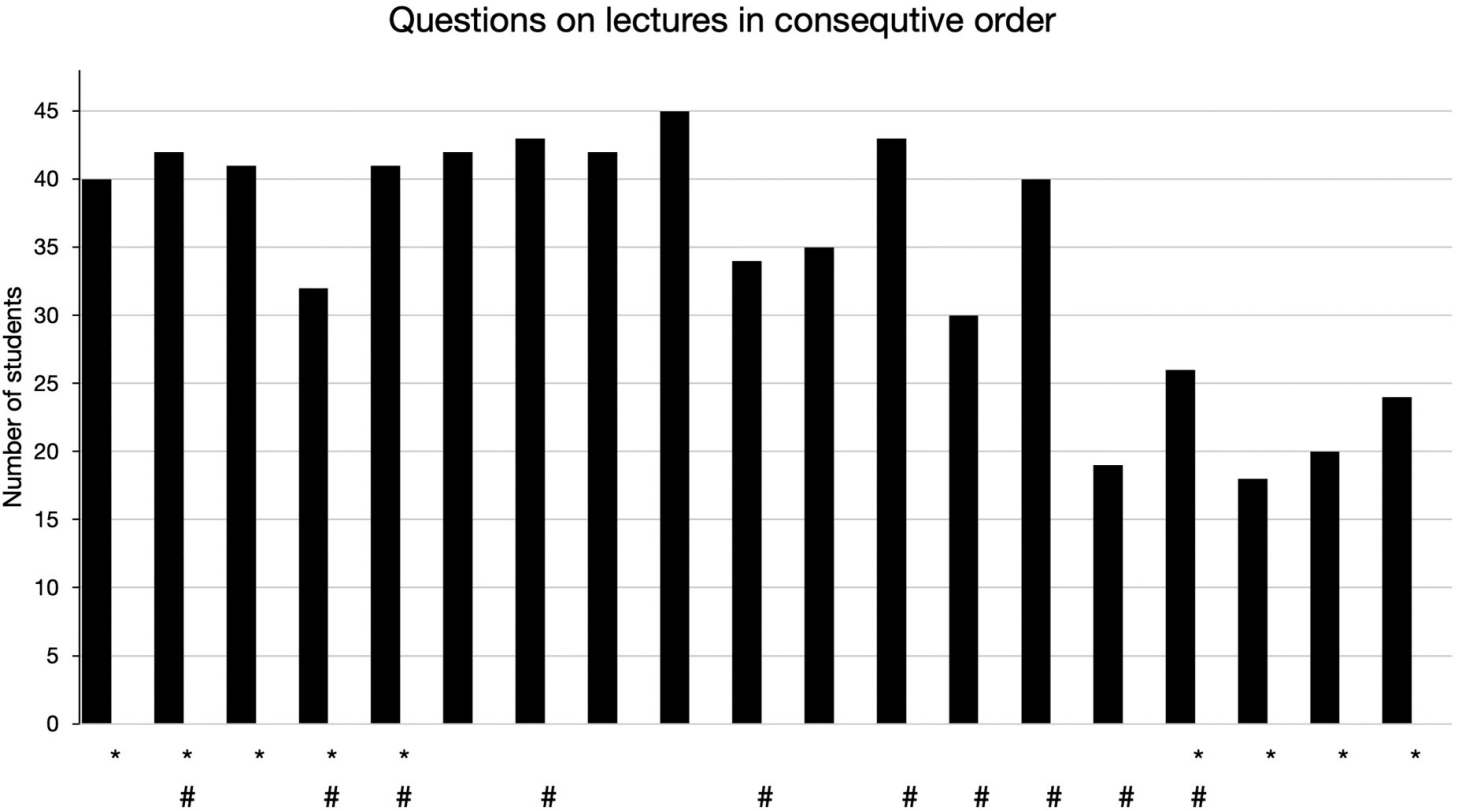
Number of students (out of 48) with a correct answer on both examination questions by lecture. *Compulsory lecture. #Remote lecture.

The distribution of examination scores for different questions, teaching delivery modes, and lecture attendance requirement deviated significantly from normality (W = 0.636-0.776, *p* < .001). No significant difference was detected between the examination scores for questions on lectures delivered in classroom and those delivered remotely (U = 175.5, *p* = .897) (Figure 2). Similarly, the distribution of examination scores was similar for the questions on the mandatory lectures and nonmandatory lectures (U = 170.0, *p* = .784). No correlation was detected between examination scores of different questions and teaching mode or lecture attendance requirement (*r_s_* = −0.022, *p* = .897 and *r_s_* = −0.048, *p* = .773). Individual students’ examination scores correlated positively with lecture attendance frequency (*r_s_* = 0.416, *p* = .004).

In total, 31 students (65% of the enrolled students) provided complete or partial responses to the end-of-course questionnaire. Table 1 displays the thematic findings. Open-ended questions were answered by 11 participants (35%) and variation of responses was observed. Majority of the respondents expressed satisfaction with the teaching mode of the course, with none considering a complete remote delivery of the course to be desirable. Of the students, 35% concluded that a blended teaching approach reduces their academic workload, while 26% felt that remote teaching would be less strenuous and 16% suggested that classroom teaching would be the preferred option.

## Discussion

The main goal of the current study was to determine whether remote teaching of undergraduate orthodontics is as effective in promoting learning as traditional classroom teaching, and to investigate whether a blended teaching approach increases students’ satisfaction. This pilot study was conducted to provide information on the study protocol for future studies aiming at orthodontic curricular development.

Previous studies, mostly conducted during the COVID-19 pandemic, among dental students, have suggested that a blended learning approach delivers an equally good learning efficiency as the traditional classroom teaching and is viewed positively by both the students and teachers.^13,20,21^ Our findings are in accordance with the previous literature from the pandemic lockdown period both in terms of learning outcome and students’ opinion.

Attendance in voluntary lectures reflects the students’ engagement with the course and their views on the quality of the teaching.^22^ Previous studies, among medical students, have reported a mean attendance of 24% to 39%, with 33% of students attending none of the nonmandatory sessions.^23,24^ Dental students have shown a tendency to attend classes more frequently as they progress in their study years; second-year dental students were more likely to be absent from classroom lectures (31%) than third-year students (15%).^25^ Female medical students have shown a better class attendance than male students.^26^ A decline from a high attendance percentage has been previously verified as a semester progresses.^24,26,27^ However, no difference in attendance at lectures that were delivered remotely as compared to in-person has been reported.^27^ The reasons most frequently given by undergraduate students for missing a lecture included boredom, illness, early morning classes, lecturer's weak presentation skills, and interference with other course work or social life.^25,28^

The evidence regarding the correlation between classroom attendance and academic performance is inconclusive, even among medical students^23,24,29–31.^ Nevertheless, a small protective effect of in-person education against stress and loneliness of medical students has been noted.^30^ Faculty and dental students have reported differing views on their perception of the relationship between attendance and academic performance; with teachers perceiving attendance at lectures being associated with improved examination performance.^32^ In the current investigation, a similar trend of decline in attendance for nonmandatory lectures was observed as the course progressed. As anticipated, the attendance was higher for mandatory lectures suggesting challenges with motivation and self-regulation. Overall, the average attendance rate was 83% and the rates for male and female students were similar. Participation in the remote and classroom lectures was comparable in the studied course, further supporting the findings of previous studies. This study identified a positive association between classroom attendance and academic performance.

As is widely acknowledged, assessment strongly influences students’ behavior and shapes their learning experience.^33^ Orthodontics at the University of Helsinki utilizes traditional written examinations, other tests, mock examinations, and objective structured clinical examinations (OSCE) for formative and summative assessment within undergraduate teaching, closely matched to the desired learning outcomes. Each course period ends with a summative examination to track student progress and provide feedback for the students, in addition to ensuring the robustness of high stakes decisions such as progression and licensing. Summative assessment also enables teachers to identify student underachievement, assess teaching effectiveness, and gather data for course improvement. Scoring essay answers consistently presents a challenge, as evidence indicates that intermarker and intramarker rating of essays is unreliable.^34^ While multiple choice questions are simpler to analyze, they pose inherent issues and may guide the respondent.^35,36^ Well-constructed context-rich multiple-choice questions are deemed to have high validity, resembling complex problem-solving exercises akin to clinical practice.^37^ The examination questions of the studied course were designed to encourage higher order thinking and reasoning skills to identify the correct answer in addition to relying on factual recall.^19^ Unlike traditional multiple-choice questions, incorrect answers may be plausible, but the correct answer was the “best” response to the clinical scenario or statement.

Dental students have been described being more satisfied with lecture-based and case-based learning delivered online than with remote teaching in problem-based learning sessions, team-based learning, and research-based learning.^38^ Reported problems of remote teaching include faculty members’ limited knowledge of technology and online teaching tools.^39^ The satisfaction scores of the first-year dental students have been found to be significantly lower than those of students with more advanced stages of their studies.^40^ The use of online simulations with targeted on-site practice has been reported as effective in clinical skills training of medical students.^41^ As pointed out by Enoch and co-workers, good online-teaching practices include synchronous and asynchronous teacher-student interactions, teamwork, and active and self-directed learning.^41^ In previous investigations, both students and lecturers have expressed a positive attitude toward developing and implementing online learning in dentistry also in the future.^21^ The advantages of digital media include an opportunity to organize lectures more flexibly and allow the students to optimize organization of their studies.^42,43^ It is important to consider which digital formats are suitable for which content in the curriculum.^42,43^ A significant variation has been found in the view on optimal amount of online teaching. While students favor an equal division between online and in-person education time, teachers state 34% to 39% time share for online education to be ideal.^20,21^ Dental students have proposed that nonclinical didactic courses, such as oral radiology, oral pathology, and basic dental sciences are best suited for distance formats, while clinical-based subjects are best delivered in person.^44,45^ Both faculty members and students agree that traditional practical training is irreplaceable and cannot be substituted entirely by remote teaching in dentistry.^21,44^ Dentistry students have been found to value the social presence and interaction that classroom-based teaching provides.^45^ Our findings are consistent with these perspectives, as none of the students preferred remote teaching as the best mode of teaching to support learning.

Several factors influence the students’ academic performance. The examined orthodontics course had 6 different lecturers, who each had their individual teaching style, and each topic taught had a different learning content which could impact student achievement. The experience of the teachers has been shown to be associated with higher examination scores.^46^ A strength of our study is that all the lecturers held PhD degrees or higher in orthodontics and had several years of experience in teaching this course.

### Limitations

A limitation of this study is the small sample size and the optional responding to the unvalidated feedback questionnaire. Data saturation was not fully reached with the themes of the qualitative questionnaire suggesting that a larger sample size or mandatory completion of the feedback questionnaire of all study participants would be beneficial. Another limitation is the nonrandom allocation of the lectures into different delivery modes. Additionally, the selection of the correct answer in SBA questions may be subject to speculation. However, the likelihood of speculation would be uniform across all questions, thereby not producing bias into our study.

Of the mandatory lectures, 44% were delivered remotely and 56% face-to-face. In total 40% of the online lectures were mandatory. The mandatory/voluntary nature of a lecture may influence students’ motivation to study a certain topic.

## Conclusions

Our finding of equal examination performance indicates, that in a blended learning course, remote teaching in orthodontics generates equally good learning outcome as classroom lectures. On average, undergraduate students favored a blended learning approach over a more traditional methodology. However, notable individual differences in opinions were observed. The insights gained from this study are valuable in developing learner-centered curricula in dentistry.

## Supplemental Material

sj-pdf-1-mde-10.1177_23821205241293488 - Supplemental material for Enhancing Dental Education: Impact of Remote Teaching on Dental Students’ Academic Performance in Orthodontics—A Pilot Study Supplemental material, sj-pdf-1-mde-10.1177_23821205241293488 for Enhancing Dental Education: Impact of Remote Teaching on Dental Students’ Academic Performance in Orthodontics—A Pilot Study by Heidi Arponen, David P. Rice and Emma Juuri in Journal of Medical Education and Curricular Development

## Data Availability

The data that support the findings of this study are available on request from the corresponding author. The data are not publicly available due to privacy restrictions.
