# Supplementary material for: Enhancing Dental Education: Impact of Remote Teaching on Dental Students’ Academic Performance in Orthodontics—A Pilot Study
Source: J Med Educ Curric Dev. 2024 Nov 3;11:23821205241293488. doi: 10.1177/23821205241293488 (PMC11533226; doi:10.1177/23821205241293488)
Supplement: sj-pdf-1-mde-10.1177_23821205241293488 - Supplemental material for Enhancing Dental Education: Impact of Remote Teaching on Dental Students’ Academic Performance in Orthodontics—A Pilot Study [file sj-pdf-1-mde-10.1177_23821205241293488.pdf]

Table 1

Standards for Quality Improvement Reporting Excellence for Education: SQUIRE-EDU

Text section and item name	SQUIRE item description	SQUIRE-EDU extension description
**Notes to authors**	The SQUIRE guidelines provide a framework for reporting new knowledge about how to improve healthcare.<br>The SQUIRE guidelines are intended for reports that describe system level work to improve the quality, safety, and value of healthcare, and used methods to establish that observed outcomes were due to the intervention(s).<br>A range of approaches exists for improving healthcare. SQUIRE may be adapted for reporting any of these.<br>Authors should consider every SQUIRE item, but it may be inappropriate or unnecessary to include every SQUIRE element in a particular manuscript.<br>The SQUIRE glossary contains definitions of many of the key words in SQUIRE.<br>The Explanation and Elaboration document provides specific examples of well-written SQUIRE items, and an in-depth explanation of each item.<br>Please cite SQUIRE when it is used to write a manuscript.	The SQUIRE-EDU extension of the SQUIRE guidelines provides a framework intended to increase the completeness, transparency, and replicability of published reports that describe systematic efforts to improve health professions education.<br>They apply to all learning settings (e.g., classroom, simulation, clinical, etc.).<br>The guidelines encourage the description of the process and context of educational change, use of iterative cycles, and use of data over time.<br>Authors should consider every SQUIRE and SQUIRE-EDU item, but it may be inappropriate or unnecessary to include every SQUIRE and SQUIRE-EDU element in a particular manuscript.<br>Not all items have an EDU extension. If there is no EDU extension, use the SQUIRE item. If there is an EDU extension, it may be used on its own or in conjunction with the SQUIRE item.<br>Educators use a range of systematic methods to make education and healthcare demonstrably better. SQUIRE-EDU may be adapted for reporting any of these methods.<br>Please cite SQUIRE-EDU when it is used to write a manuscript.

**Title and abstract**		
1. Title	Indicate that the manuscript concerns an initiative to improve healthcare (broadly defined to include the quality, safety, effectiveness, patient-centeredness, timeliness, cost, efficiency, and equity of healthcare)	**EDU 1:** Indicate that the manuscript concerns efforts to improve health professions education systems and learning<br>page 1
2. Abstract	a. Provide adequate information to aid in searching and indexing<br>b. Summarize all key information from various sections of the text using the abstract format of the intended publication or a structured summary such as: background, local problem, methods, interventions, results, conclusions	**EDU 2:** Keywords include a focus on education and learning<br>page 2
**Introduction: Why did you start?**		
3. Problem description	Nature and significance of the local problem	**EDU 3:** Description of the nature and significance of the need for change in the local educational system page 3
4. Available knowledge	Summary of what is currently known about the problem, including relevant previous studies	--
5. Rationale	Informal or formal frameworks, models, concepts, and/or theories used to explain the problem, any reasons or assumptions that were used to develop the intervention(s), and reasons why the intervention(s) was expected to work	**EDU 5:** Identify the guiding theory (learning, change, implementation, or other) and how it aligns with the need for change in the local educational system<br>page 3-4
6. Specific aims	Purpose of the project and of this report	-- page 4
**Methods: What did you do?**		
7. Context	Contextual elements considered important at the outset of introducing the intervention(s)	**EDU 7a:** Contextual elements for learning (*e.g.*, setting, program, people, resources, social, geopolitical influences) before the intervention(s) page 5-6<br>**EDU 7b:** The interrelationships between the contextual elements and the local educational

8. Intervention(s)	a. Description of the intervention(s) in sufficient detail that others could reproduce it<br>b. Specifics of the team involved in the work	**EDU 8a:** Description of the primary interventions and co-interventions (e.g., faculty or tool development)<br>**EDU 8b:** Specify how the interprofessional education team (e.g., faculty, staff, patients, and learners) was part of the design of the intervention	page 6<br>page 6
9. Study of the intervention(s)	a. Approach chosen for assessing the impact of the intervention(s)<br>b. Approach used to establish whether the observed outcomes were due to the intervention(s)	**EDU 9a:** Approach used to understand the impact of the educational intervention(s) on the learner and beyond, such as impact on patients, families, the community, faculty, educational program, or the healthcare system<br>**EDU 9b**: Approach to assess the fidelity of and the iterative changes to the planned intervention(s) over time	page 7<br>Not applicable, pilot study
10. Measures	a. Measures chosen for studying processes and outcomes of the intervention(s), including rationale for choosing them, their operational definitions, and their validity and reliability<br>b. Description of the approach to the ongoing assessment of contextual elements that contributed to the success, failure, efficiency, and cost<br>c. Methods employed for assessing completeness and accuracy of data	**EDU 10:** Quantitative and/or qualitative measures chosen to assess the educational processes and outcomes on learners, faculty, educational programs, patients, families, healthcare systems, or communities	page 7-8
11. Analysis	a. Qualitative and quantitative methods used to draw inferences from the data<br>b. Methods for understanding variation within the data, including the effects of time as a variable	--	page 7
12. Ethical considerations	Ethical aspects of implementing and studying the intervention(s) and how they were addressed, including, but not limited to, formal ethics review and potential conflict(s) of interest	**EDU 12:** Approaches to address vulnerability of learner participants	page14-15
**Results: What did you find?**			

13. Results	a. Initial steps of the intervention(s) and their evolution over time (e.g., time-line diagram, flow chart, or table), including modifications made to the intervention during the project<br>b. Details of the process measures and outcome<br>c. Contextual elements that interacted with the intervention(s)<br>d. Observed associations between outcomes, interventions, and relevant contextual elements<br>e. Unintended consequences such as unexpected benefits, problems, failures, or costs associated with the intervention(s).<br>f. Details about missing data	**EDU 13a:** For each educational intervention and co-intervention, provide details about iterative modifications based on the assessment of the learning	Not-Applicable<br>page 7
**Discussion: What does it mean?**			
14. Summary	a. Key findings, including relevance to the rationale and specific aims<br>b. Particular strengths of the project	**EDU 14:** Connect the findings to the guiding theory (learning, change, implementation, other) used to direct the change in the local educational system	page 10
15. Interpretation	a. Nature of the association between the intervention(s) and the outcomes<br>b. Comparison of results with findings from other publications<br>c. Impact of the project on people and systems<br>d. Reasons for any differences between observed and anticipated outcomes, including the influence of context<br>e. Costs and strategic trade-offs, including opportunity costs	**EDU 15c:** Include the impact of the intervention(s) on learners, faculty, educational program, patients, families, healthcare systems, or communities	pages 9-14
16. Limitations	a. Limits to the generalizability of the work<br>b. Factors that might have limited internal validity such as confounding, bias, or imprecision in the design, methods, measurement, or analysis<br>c. Efforts made to minimize and adjust for limitations	--	page 14

17. Conclusions	a. Usefulness of the work<br>b. Sustainability<br>c. Potential for spread to other contexts<br>d. Implications for practice and for further study in the field<br>e. Suggested next steps	**EDU 17b:** Scalability of the work to other learners and contexts<br>**EDU 17d:** Lessons learned for clinical practice, education, and policy	page 14
**Other information**			
18. Funding	Sources of funding that supported this work. Role, if any, of the funding organization in the design, implementation, interpretation, and reporting	--	page 15

Table 2

Example and Explanation of Each SQUIRE-EDU (Standards for Quality Improvement Reporting Excellence for Education) Item[a]

SQUIRE-EDU item	Example of text in a manuscript	Comments
**EDU 1.** Indicate that the manuscript concerns efforts to improve health professions education systems and learning	Student-Focused Improvements in a Preclinical Medical Physiology Course	--
**EDU 2.** Keywords include a focus on education and learning	medical education, physiology, educational improvement, flipped classroom	--
**EDU 3.** Description of the nature and significance of the need for change in the local educational system	Based on student feedback and national trends, the medical school preclinical physiology course started a multi-year transformation to a flipped classroom experience. This shift was initiated because of low student satisfaction from the lecture-based class structure.	The course director undertook a major, iterative redesign because the current structure was not meeting the needs of the students. Student dissatisfaction is the local gap that was addressed using educational improvement.
**EDU 5.** Identify the guiding theory (learning, change, implementation, or other) and how it aligns with the need for change in the local educational system	Adult Learning Theory supports that by flipping the pedagogy to a student-centered model, medical students will develop a deeper understanding of the material and be more able to apply their learning to patient care.	This statement encompasses both the rationale for the change as well as the learning theory (adult learning theory) that were key components of the design of this educational improvement project.
**EDU 7a.** Contextual elements for learning (e.g., setting, program, people, resources, social, geopolitical influences) before the intervention(s)	Physiology is one of eight foundational science courses at the SOM. Each course is led by a faculty member who has oversight of the course and leads approximately 75% of the sessions. For the past 12 years, the course has used mostly large group lecture format.	This description gives the reader a sense of the education program structure, the recent history of leadership in the course, volume of students, and relationship of the course to external influences, such as the dean and the accreditation committee. A reader gets a sense of what the authors believed to be important in starting the work to improve the physiology course and can determine how similar or different it is to his or her own context.
**EDU 7b.** The interrelationships between the contextual elements and the	A new course director was named in 2014. The SOM matriculates 125 students each year and recently completed its accreditation visit. Although it received	

local educational and healthcare systems before the intervention(s)	full accreditation, the dean noted that the preponderance of lecture style teaching was a vulnerability noted by the accreditation committee and encouraged faculty to update pedagogy.	
**EDU 8a.** Description of the primary interventions and co-interventions (e.g., faculty or tool development)	The physiology course is divided into four different sections. The respiratory physiology section leader created the initial flipped classroom experience in 2014 by making online recordings of the lectures from the previous academic year.	This section describes the initial planned change with a recognition that most curricular changes require multiple interventions.
**EDU 8b.** Specify how the interprofessional education team (e.g., faculty, staff, patients, and learners) was part of the design of the intervention	Students' evaluation provided data that they wanted more pre-work prior to class, so to use class time in a more interactive way. The lecture recordings were processed by the course assistants to be used as the pre-work for students to watch prior to class so that they could work on respiratory physiology questions and problems in groups during the class session. Instructional designers and faculty collaborated to build the in class sessions.	In educational improvement, it is common to have students feedback serve as a catalyst for initiating improvement. Other input, such as care gaps identified by patients or families would be included in this section. Specify the specific individuals who helped to build the interventions.
**EDU 9a.** Approach used to understand the impact of the educational intervention(s) on the learner and beyond, such as impact on patients, families, the community, faculty, educational program, or the healthcare system	In order to monitor the development of the changes to the course, four students who were enrolled in the course were recruited each year to gather field notes. After training to ensure reliable data gathering, the students wrote down observations and reflections about the design and execution of the flipped classroom activities. The students wrote these anonymously during the course. Field notes were not part of the students' grade and were important data that were assessed after grades were finalized.	This is one example of how a team might study the intervention while the intervention is being implemented. By enlisting students to provide detailed feedback, the teaching team is able to gather first-hand information about the efficacy of the intervention. This approach differs from course feedback that occurs subsequent to the intervention and may only provide insight into satisfaction with the learning experience.
**EDU 9b.** Approach to assess the fidelity of and the iterative changes to the planned intervention(s) over time	Data from the student field notes were used to modify the flipped classroom experience in each subsequent iteration.	Field notes are one way to gather detailed, deep, and real time data about a change process.

**EDU 10.** Quantitative and/or qualitative measures chosen to assess the educational processes and outcomes on learners, faculty, educational programs, patients, families, healthcare systems, or communities	The physiology course assessed the changes to the curriculum across several domains. Achievement in physiology was measured with scores on the written quizzes, final exam, and on USMLE Step 1 physiology scores. Student satisfaction was assessed through the usual course feedback system. Field notes from selected students tracked the impact and fidelity of the iterative changes to the curriculum. Finally, impact on patients was assessed through an observed structured simulation exercise having students react to physiologic changes in high fidelity mannequins.	The variety of data measures in the example allow the teaching team to understand the breadth of impact of the educational changes. A reader of the manuscript will immediately be able to understand whether the changes relate to their own context or not.
**EDU 12.** Approaches to address vulnerability of learner participants	Improvements in the physiology course were reviewed and approved by the local IRB. Additionally, student participation in gathering field notes was voluntary and clearly communicated as not connected to students' grades in the physiology course.	Learners are a vulnerable population and require adequate protection from the appropriate institutional ethics review committee. Addressing the perception of coercion is an important element of ethical conduct of educational improvement.
**EDU 13a.** For each educational intervention and co-intervention, provide details about iterative modifications based on the assessment of the learning	See Table 3. Example of Iterations to the Physiology Course, 2014-17	A table such as the example is not the sole outcome but is intended to complement other identified outcomes.
**EDU 14.** Connect the findings to the guiding theory (learning, change, implementation, other) used to direct the change in the local educational system	Active learning theory and data guided the iterations of the interventions. While this appeared straightforward at the outset, many unforeseen consequences occurred as the changes were made over the four years. First, faculty development was paramount, and faculty required boosters along the way. As the faculty became more comfortable with the flipped teaching method, the sessions became smoother. Second, students preferred the active learning, but only to a point. Students expressed a desire for a combination of lectures and active learning. These requests were unexpected for the	In the example, there is no change in knowledge assessment, some unexpected results from student reactions, and promising application of the knowledge. Together, these findings provide a comprehensive assessment of the development and changes made to the physiology course.

	teaching team, but no less important to address in future iterations.	
**EDU 15c.** Include the impact of the intervention(s) on learners, faculty, educational program, patients, families, healthcare systems, or communities	Student performance on exams and USMLE national board exams were unchanged. Students performed very well on the simulation exercise, indicating that they were capable of learning complex respiratory and renal physiology and able to apply it in a patient setting. This application of knowledge was a significant step forward for the physiology course and will be explored further in the curriculum.	Performance on USMLE was one important data outcome that was carefully tracked. The physiology course team also created a specific assessment (integrated high-fidelity simulation) to determine how the new teaching method might impact patient care. Keeping in mind the impact of health professions education is ultimately on the care provided, this outcome tried to assess the potential effect of the improved physiology course.
**EDU 17b.** Scalability of the work to other learners and contexts	Although the improvement s in physiology was quite successful over four years, the school has struggled to make similar active learning advances in other courses. The mandate from the dean to increase active learning led to some starts and stops in other courses, but not the sustained effort that was seen in Physiology.	Perhaps included in this section might be the perceived barriers to adoption of the innovative pedagogy in other parts of the curriculum.
**EDU 17d.** Lessons learned for clinical practice, education, and policy	Others would be wise to ensure that a comprehensive evaluation system is in place so that the teaching team can react to and modify the curriculum as needed. Also, the connection to the integrated physiology simulation assessment created an important motivator for the students. This connection to "real" patient care was vital for the success of this project.	Describing the potential next phases of this work through the "lessons learned" will guide subsequent iterations of educational improvement for the readers.

Abbreviations: SOM indicates school of medicine; IRB, institutional review board; USMLE, United States Medical Licensing Examination.

[a]This is a fictitious example we have created from a preclinical, medical school physiology course that systematically changed its teaching and evaluation over several annual cycles. This example is intended to demonstrate how educational improvement can be addressed in a manuscript using SQUIRE-EDU.

Table 3

Example of Iterative Changes to a Physiology Course, 2014–2017 Using SQUIRE-EDU (Standards for Quality Improvement Reporting Excellence for Education)[a]

Year	Changes made	Outcomes	Lessons learned
2014	5 of 15 respiratory physiology 1-hour lectures replaced with flipped classroom	• Data from field notes indicated prework too long<br>• Data from field notes indicated classroom sessions needed to be much more interactive	• Prework more focused<br>• Increase difficulty of classroom activities
2015	10 of 15 respiratory physiology 1-hour lectures replaced with flipped classroom	• Test results indicate prework was focused better<br>• Data from field notes indicate that classroom sessions were a bit too difficult<br>• Scores on exams unchanged from historical controls	• Spread to renal physiology section<br>• Try a simulation of respiratory physiology for the students assessment
2016	5 of 15 respiratory physiology 1-hour lectures replaced with flipped classroom<br><br>5 of 15 renal physiology 1-hour lectures replaced with flipped classroom	• Student evaluation data indicated that prework was acceptable and focused<br>• Data from field notes indicate that classroom sessions interactive and challenging, but renal faculty struggled with the format<br>• Exam scores increased, but not significantly<br>• Data from faculty running simulation indicate that simulation was challenging	• Faculty development for renal physiology faculty regarding teaching methodology<br>• Expand simulation to include combined respiratory and renal physiology
2017	All respiratory and renal physiology lectures replaced with flipped classroom activities	• Data from student field notes suggest that classroom sessions were interactive and challenging	• Add in foundational content knowledge to assist students to be successful in the classroom sessions

	• Data from faculty indicate that renal faculty better prepared for facilitating the classroom sessions<br>• Data from student evaluation include student requests for foundational lectures<br>• Data from class evaluation indicate that simulation shows promise as interactive and challenging final exam	• Add students to the course evaluation team<br>• Sharpen the simulation examination

[a]This accompanies Table 2 from the fictitious example from a preclinical, medical school physiology course that systematically changed its teaching and evaluation over several annual cycles. This table is an example of how the iterative changes to a course might be chronicled in a manuscript using SQUIRE EDU.
